# Fluorescent imaging of the biliary tract during laparoscopic cholecystectomy

**DOI:** 10.1186/s13022-014-0005-7

**Published:** 2014-08-12

**Authors:** Darren Leonard Scroggie, Claire Jones

**Affiliations:** 1 UCL Division of Surgery & Interventional Science, 9th Floor, Royal Free Hospital, Pond Street, London NW3 2QG, United Kingdom; 2 Department of HPB Surgery, Mater Infirmorum Hospital, Crumlin Road, Belfast BT14 6AB, United Kingdom

**Keywords:** Fluorescent cholangiography, Laparoscopic cholecystectomy, Intraoperative cholangiography, Imaging, Fluorophore

## Abstract

The introduction of laparoscopic cholecystectomy was associated with increased incidences of bile duct injury. The primary cause appears to be misidentification of the biliary anatomy. Routine intra-operative cholangiography has been recommended to reduce accidental duct injury, although in practice it is more often reserved for selected cases. There has been interest in the use of fluorescent agents excreted via the biliary system to enable real-time intra-operative imaging, to aid the laparoscopic surgeon in correctly interpreting the anatomy. The primary aim of this review is to evaluate the ability of fluorescent cholangiography to identify important biliary anatomy intra-operatively. Secondary aims are to investigate its ability to detect important intra-operative pathology such as bile leaks, identify potential alternative fluorophores, and evaluate the evidence regarding patient outcomes.

## Introduction

Misidentification of the biliary anatomy has been cited as the commonest cause of bile duct injury during laparoscopic cholecystectomy [1]. Although this complication is infrequent [2], affected patients suffer considerably [3]. Therefore attempts to further reduce its incidence are justifiable. Intra-operative cholangiography has been recommended to reduce bile duct injuries, although in practice it is often reserved for selected cases [4]. Fluorescent cholangiography (FC) is a novel technique which offers real-time intra-operative imaging of the biliary anatomy. The primary aim of this review is to evaluate the ability of FC to identify important biliary anatomy intra-operatively. Secondary aims are to investigate its ability to detect important intra-operative pathology such as bile leaks, identify potential alternative fluorophores, and evaluate the evidence regarding patient outcomes.

**Figure 1:**
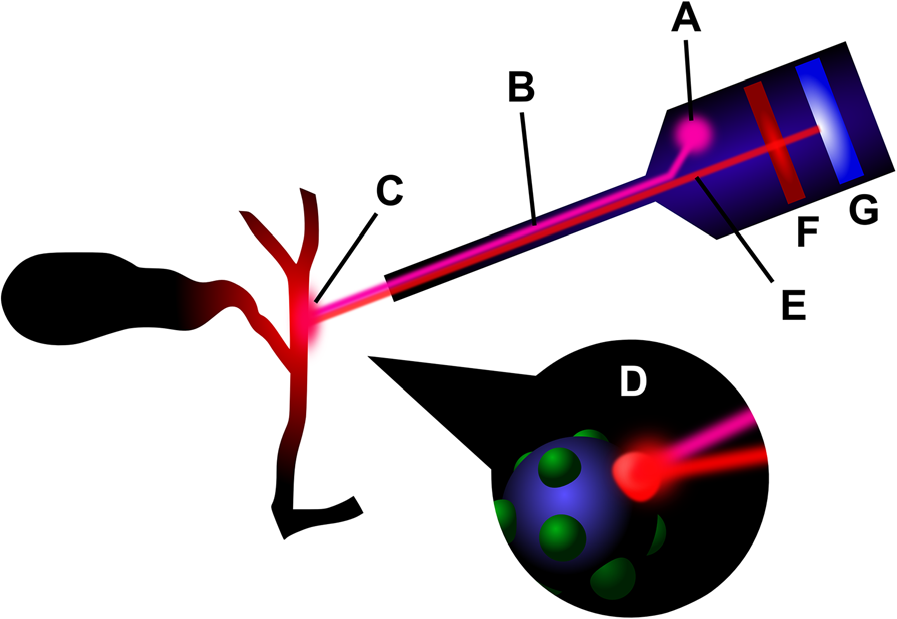
**Principle of fluorescent cholangiography.** A source of near-infrared light **(A)** emits an excitation wave **(B)**, which is directed towards the fluorophore-filled biliary tree at **(C)**. The fluorophore, shown bound to a protein, is excited **(D)**, causing emission of a longer wavelength **(E)**. A filter **(F)** removes unwanted shorter wavelengths. An image of the fluorescing biliary tree is formed on the charge-coupled device **(G)**, which is then processed for viewing by suitable electronics.

The first intra-operative use of FC in humans was described by Ishizawa *et al*. [5]. The method involved the administration of indocyanine green (ICG) by either intra-biliary injection, or intravenous injection before surgery. ICG binds to proteins present in bile, and is excreted exclusively by the liver when administered intravenously. The excitation of protein-bound ICG by near-infrared light causes it to fluoresce, thereby delineating components of the biliary system to the surgeon. This is accomplished by the use of a specialised camera system, which illuminates the target with near-infrared light and filters the reflected wavelengths, such that the fluorescing ICG is clearly observed. The principle of FC is illustrated in Figure 1.

Intra-operative FC has been successfully performed during laparoscopic cholecystectomy in several studies [6]-[14]. These include standard multiple-incision, single-incision and robot-assisted single-incision operations. The technique is not limited to use during laparoscopic cholecystectomy only; its feasibility and utility during hepatectomies for tumours and living donor liver transplantation have also been described [15]. It has been used in sentinel lymph node mapping during breast cancer surgery, prior to its adoption for FC [16].

Various devices have been described for use in FC. They represent different physical realisations of the same basic principles. Common to all these devices is the inclusion of an excitation light source to cause fluorescence, and a charge coupled device (CCD) with filtering to capture the fluorescent image. A method of switching between the normal viewing mode and the fluorescence mode is provided in the case of laparoscopic cameras. Differences exist in the physical forms of the devices, the excitation wavelength, the emission filter, and the image capture characteristics of the CCD. The device used by Ishizawa *et al.*[5] during open cholecystectomies and liver resections was a hand-held unit with a separate control unit. Light-emitting diodes produced near-infrared excitation at 760 nm, and the image was captured by the CCD camera after filtering out wavelengths shorter than 820 nm. The fluorescence was detectable by the CCD as excited ICG emits a longer wavelength of about 830 nm. This was viewable both by the naked eye and on a television monitor with the operating lights off. This device was intended for open surgery, and was not suitable for laparoscopic use. A different device developed for sentinel lymph node mapping in breast cancer surgery [17] has been successfully used during open hepatobiliary surgery [14].

Ishizawa *et al.* later report successfully performing ICG FC during laparoscopic cholecystectomy, using a different prototype device [18]. The described instrument comprised a xenon light source, a small control unit, and a laparoscope with CCD camera filtering wavelengths below 810 nm. A foot switch allowed the surgeon to alternate between standard and fluorescent images. The practicality of the same or similar systems in laparoscopic cholecystectomy has been verified in several other studies; a device for use with the da Vinci robot is also available, and robot-assisted laparoscopic cholecystectomy with ICG fluorescent cholangiography has been performed in many patients [7],[10],[11].

## Review

### Identification of biliary structures

The first human study of intra-operative FC in open surgery reported the successful identification of several important biliary structures [5]. Intra-biliary injection of ICG allowed identification of the common hepatic duct (CHD) and confluence of the right and left hepatic ducts in 100% of patients (n = 13) undergoing hepatectomy. Intravenous administration in patients (n = 10) undergoing open cholecystectomy revealed the CHD in 100%, and the cystic duct (CD) in 90%. Visualisation of the common bile duct (CBD) is not reported in this study.

The use of the laparoscopic system also appears to effectively delineate extrahepatic ducts. The structures successfully identified before dissection of Calot’s triangle include the CD, CHD, CBD, and the junction of the CD with the CHD [6]-[10],[12],[19]. These include standard, single-incision and robotic procedures. Table 1 summarises the published detection rates of major biliary structures before dissection of Calot’s triangle. Overall, the preliminary studies suggest the technique is effective in identifying important biliary anatomy. However, significant differences in technique and methodology exist between studies; therefore it is difficult to accurately quantify overall rates of detection. Furthermore, it is not apparent whether or not the surgeon could have identified the same structures unaided.

**Table 1:** Detection rates of biliary structures using fluorescent cholangiography

**Study**	**Technique**	**N**	**CD**	**CHD**	**CD/CHD junction**	**CBD**
Ishizawa et al. (2010) [12]	LC	52	52 (100%)	50 (96.2%)	50 (96.2%)	-
Aoki et al. (2010) [19]	LC	14	10 (71.4%)	-	-	10 (71.4%)
Ishizawa et al. (2011) [6]	SILC	7	5 (71.4%)	7 (100%)	7 (100%)	-
Kaneko et al. (2012) [8]	LC	28	26 (92.9%)	27 (96.4%)	-	-
Buchs et al. (2012) [7]	SIRC	12	11 (91.6%)	4 (33.3%)	3 (25%)	6 (50%)
Schols et al. (2013) [9]	LC	15	15 (100%)	-	-	15 (100%)
Spinoglio et al. (2013) [10]	SIRC	45	42 (93%)	40 (80%)	40 (80%)	41 (91%)

LC, standard laparoscopic cholecystectomy.

SILC, single-incision laparoscopic cholecystectomy.

SIRC, single-incision robotic cholecystectomy.

N, number of patients in study.

-, data not reported.

The literature contains some reports of FC identifying anatomical variants of the biliary tree. In a study of open surgery, it effectively identified right lateral sector branches draining into the CHD [5]. A study of the laparoscopic technique demonstrated a sensitivity of 100% for detecting accessory hepatic ducts in 8 patients, after dissection of Calot’s triangle [12]. In these patients, such variants had already been identified during pre-operative investigations. A parallel course of the CD and CHD was identified in one laparoscopic study using FC [9]. There are no reports of detection of anatomical variants during robotic cholecystectomy. As yet, there are no studies to determine the sensitivity of FC in detecting anatomical variants whose existences are not already known from prior investigations.

An extension of the fluorescence technique has been described to enable intra-operative fluorescent angiography alongside cholangiography, for the purpose of identifying the cystic artery [8],[13]. The technique involves a pre-operative intravenous injection of ICG to facilitate FC, with a second ICG bolus administered intra-operatively to enable identification of the cystic artery. In the larger of the studies, the cystic artery began to fluoresce at 20 to 30 seconds after the bolus, and was identified successfully in 25 of 28 patients (89.3%) [8]. Fluorescence of the artery lasted for over 5 to 10 seconds. The authors suggest this ICG re-injection technique may facilitate safer dissection of the cystic artery, especially in difficult cases.

FC has several specific limitations described. The major one is its inability to visualise deep intrahepatic ducts, or extrahepatic ducts covered with surrounding organs and tissue, due to the limited tissue penetration of near-infrared light [5]. It can be unusable in obese patients [19]. Calot’s triangle needs to be adequately exposed to obtain good fluorescent images, which can disadvantage the inexperienced laparoscopic surgeon [12]. A simple transparent plastic compression device has been described to help overcome these problems, which can reduce the thickness of extramural tissue and facilitate a clearer view [13].

The other limitations are less clear due to lack of good quality supporting evidence. Its ability to detect biliary stones, bile leaks and bile duct injuries in humans is not known. No reports investigate user dependence. It is not clear if FC affects the operative time, although a recent study of its use during robotic laparoscopic cholecystectomy found no statistically significant difference compared to standard robotic laparoscopic cholecystectomy [11]. If it is to be pursued for these various applications, more research in large animals or humans is required.

### Detection of bile leaks

The ability of FC to detect intra-operative bile leaks has been investigated to a limited extent. Studies in mouse models have shown that leaks were more apparent on fluorescent images than on standard imaging [20],[21]. Bile leaks were readily identified even without a fluorophore, due to the autofluorescence of bile [21]. Tagaya *et al.* observed that bile leakage caused by cannulation of the cystic duct in humans for IOC was easily visualised on fluorescent imaging [13]. However, detection of previously unknown bile leaks has not been reported.

### Detection of biliary stones

An important application of standard fluoroscopic intra-operative cholangiography (IOC) is the detection of biliary stones. It has been shown in mouse models that simulated CBD stones can be imaged using fluorescence of VM674 [21]. There is no evidence that FC can effectively identify CBD stones in humans. Most of the human studies lack patients with CBD stones. One study describes a failure to identify a CBD stone in one patient which had previously been identified during pre-operative investigations, although CD stones were identified in some patients [12]. The ability of the technique to detect stones elsewhere in the biliary tree, such as the CHD, has not been investigated. Indeed, the intra-hepatic ducts and those covered by thick tissue are inaccessible [5]. Thus the available evidence does not suggest that FC is likely to replace standard intra-operative cholangiography in cases where biliary stones are suspected.

### Fluorophores

ICG is the only agent that is reported to have been used in humans for the purpose of FC. There has been a controlled trial in mouse models of VM674, a fluorophore designed and developed for rapid biliary excretion after intravenous administration [21]. This study demonstrated rapid and sustained visualisation of the biliary anatomy, and image target-to-background ratios favouring VM674 over ICG. However, proponents of ICG argue that the VM674 technique may not work so well in humans; the shorter wavelengths of light used for VM674 have poorer tissue penetration, and the connective tissue around the biliary system in humans is much thicker compared to that of the mouse [22]. The pharmacokinetic properties of VM674, particularly rapid and high biliary excretion, may make it more suitable for imaging acute biliary injuries or leaks that develop intra-operatively, as ICG needs to be administered well in advance [23].

Other fluorophores have been investigated in rats and pigs, notably an ICG-related molecule called CW800-CA which produces a higher signal-to-background ratio than ICG [24]. ICG remains the only fluorophore to be used in humans due to its established safety and availability, although the animal studies suggest it may not necessarily be the most efficient. It has also been demonstrated in mice that FC can be performed without the aid of a fluorophore; bile will autofluoresce when excited by light at 475 nanometres, sufficiently intensely as to render a real-time cholangiogram [20].

The equipment used for non-ICG fluorophores differs in the wavelengths of light involved. For VM674, an excitation wavelength of 649 nm is used, producing fluorescence at 675 nm [21]. In the autofluorescence technique, the excitation is 475 nm, producing emissions at 480 nm [20]. Both of these experimental studies involved mouse models, using appropriate equipment. These techniques have not been applied to humans, and no suitable equipment is described.

The timing of administration of the fluorophore merits consideration. ICG injected into the bile duct is administered intra-operatively, whereas intravenous ICG is administered pre-operatively [5]. There is a delay from intravenous injection until the extrahepatic ducts become visible by fluorescence. This time interval has been quantified at 90 minutes in pig models [25]. Studies in humans report satisfactory results with administration 30 minutes or one hour before surgery, with fluorescence lasting at least until closure of the abdominal incisions [19]. A recent study investigating the dose and timing of ICG administration found that a prolonged interval of 24 hours between administration and surgery produced optimal contrast of the CBD against the liver, compared to administration 30 minutes before surgery [14]. The improved contrast was due to a lower liver background signal. However, intervals between the two extremes were not investigated. Furthermore, the excretion of intravenously administered ICG is impaired by hepatic dysfunction [26].

The alternative fluorophore VM674 is more rapidly excreted in bile, and the extrahepatic ducts in mouse models can be identified by fluorescence between 2 and 5 minutes after intravenous injection, with a fluorescence peak at 25 minutes [21]. However, this property of VM674 has not been investigated in humans. Therefore, there is a requirement for more research to determine the optimal administration protocols for the fluorophore.

The effect of the fluorophore dose has been investigated in humans using ICG [14]. It appears from this study that larger doses of ICG cause more fluorescence of the liver, which reduces the signal-to-background ratio of the bile ducts. Since both the ICG dose and time interval from administration to surgery affected the signal-to-background ratio, the optimum dose needs to be matched to this time interval. Unfortunately the study only investigates doses of 5 mg, 10 mg and 20 mg, and time intervals of 30 minutes and 24 hours. The optimal dose and time may lie between these values.

### Patient outcomes

Whilst FC may afford the surgeon the ability to more readily define the extrahepatic biliary anatomy, it is of more importance to consider its effect on the patient. Firstly, it is logical to consider the effect of the fluorophore itself on the patient. ICG is already in routine clinical use and is considered generally safe, but adverse reactions have been reported. A review by Benya *et al.* described the various reactions that have been reported [27]. In this group of patients, urticaria, sensations of warmth and headache were common. Dyspnoea and wheezing are also reported, and one patient died due to laryngospasm. Other reactions include sore throat, nausea, pruritis, peripheral vasodilatation with hypotension, tachycardia, pulmonary congestion, and oedema, although overall reactions are rare [28]. Therefore concerns of adverse reactions are not sufficient to preclude the use of ICG in routine laparoscopic cholecystectomy.

It is not yet clear whether improved visualisation of the extrahepatic biliary anatomy by FC actually translates into improved patient outcomes. Although no bile duct injuries have been reported when using FC, the sample sizes are small and the studies may be inadequately powered to detect rare outcomes. The largest study of FC in laparoscopic cholecystectomy enrolled 52 patients [12]. The median post-operative hospital stay was 5 days, and no postoperative bile leaks occurred. It is not apparent why the hospital stays reported in this study are considerably longer than those reported in other studies of laparoscopic cholecystectomy. A recent trial of laparoscopic cholecystectomy using different numbers of incisions describes a mean post-operative hospital stay of only 1.2 days for a 4-port technique [29]. There is similarly a lack of data on longer term outcomes, as the available studies focus primarily on the intra-operative period, with brief mentions of the early post-operative period. It is necessary to investigate the need for re-admission and re-operation after discharge, in order to determine whether FC has any beneficial influence on these longer-term outcomes.

## Conclusion

FC is a novel imaging technique which has been shown to effectively illuminate the extrahepatic biliary anatomy during laparoscopic surgery, producing impressive detection rates for the more important structures. The most extensively used fluorophore, ICG, is already clinically available. To date, human studies of FC in laparoscopic cholecystectomy have demonstrated its feasibility and apparent safety. It is unlikely to replace fluoroscopic IOC in cases where biliary stones are suspected, and there is insufficient evidence as yet to determine its impact on important adverse events associated with laparoscopic cholecystectomy. Nevertheless its relative simplicity may justify its use if it can be shown to reduce bile duct injuries. Further research should aim to quantify the effects of this technique on adverse events and long-term patient outcomes.

## Abbreviations

CBD: Common bile duct

CCD: Charge-coupled device

CD: Cystic duct

CHD: Common hepatic duct

FC: Fluorescent cholangiography

ICG: Indocyanine green

IOC: Intra-operative cholangiography

## Competing interests

The authors declare that they have no competing interests.

## Authors’ contributions

DS conceived the article and conducted the initial literature search. DS and CJ produced the final version. Both authors have read and approved the manuscript.

## Authors’ information

DS is a surgical trainee in the United Kingdom. He is a Member of the Royal College of Surgeons of Edinburgh, and is currently completing an MSc degree in surgical science at University College London.

CJ is an ST7 surgical trainee in general & hepatopancreaticobiliary surgery in the United Kingdom. She is a Fellow of the Royal College of Surgeons of Edinburgh, and holds MPhil and MSc degrees from Queen’s University Belfast and Cardiff University respectively.
